# New Developments in the Treatment of Neuroendocrine Tumours – RADIANT-4, NETTER-1 and Telotristat Etiprate

**DOI:** 10.17925/EE.2016.12.01.44

**Published:** 2016-03-15

**Authors:** Emilia Sbardella, Ashley Grossman

**Affiliations:** Department of Endocrinology, Oxford Centre for Diabetes, Endocrinology and Metabolism, Churchill Hospital, University of Oxford, Oxford, UK

**Keywords:** Neuroendocrine tumours, treatment, everolimus, ^177^Lu-DOTATATE, 5-HT synthesis inhibitor, telotristat etiprate

## Abstract

Neuroendocrine tumours (NETs) are a heterogeneous group of neoplasms whose incidence has increased significantly in recent years, and whose optimal management remains controversial. We report the latest innovations in their management, in particular the results of three trials concerning the use of the mammalian target of rapamycin (mTOR) inhibitor, everolimus, in non-functional NETs of lung/ gastrointestinal (GI) origin, the first randomised trial of radiolabelled ^177^Lu-DOTATATE in patients with mid-gut NETs, and the use of the 5-HT synthesis inhibitor, telotristat etiprate, in patients with the carcinoid syndrome.

Neuroendocrine tumours (NETs) represent a heterogeneous group of neoplasms that originate from different types of neuroendocrine cells throughout the body.^1^ While previously considered to be relatively uncommon, their overall incidence has been reported as increasing for reasons which are unclear.^2–4^ Surgery is the only truly curative therapy, but there are now a variety of other treatment options although their specific use and sequencing remains controversial.^3^ However, somatostatin analogues (SSAs), particularly the long-acting formulations of octreotide and lanreotide, are highly effective for symptomatic patients with secretory syndromes, and recent studies have also shown their ability to retard tumour progression in the PROMID, CLARINET and RADIANT-2 trials.^5–8^ The multi-ligand SSA pasireotide was also shown to control the symptoms of carcinoid syndrome in patients with advanced NETs refractory/resistant to octreotide long-acting release (LAR) therapy.^9^ Recently, at the European Cancer Congress (ECC) of the European Society for Medical Oncology (ESMO) in Vienna, in September 2015, three trials were presented which provide novel data on therapeutic options.

Previous studies have demonstrated the efficacy of everolimus, an inhibitor of mammalian target of rapamycin (mTOR) (a serine-threonine kinase that stimulates cell growth, proliferation and angiogenesis)^10–12^ to slow tumour progression of pancreatic NETs (RADIANT-3)^13^ and symptomatic mid-gut tumours (RADIANT-2).^7^ RADIANT-4 was a placebo-controlled, double-blind, phase III study carried out in 13 European centres on the efficacy and safety of everolimus in patients with advanced, progressive, non-functional NETs of the lung and gut.^14^ Non-functional NETs are often diagnosed later when the cancer has become advanced, and at present there are limited treatment options available. This is particularly important for patients with lung carcinoids, as there is currently no approved treatment for such patients.The trial included 302 patients in which the patients were randomised (2:1) to everolimus (10 mg/d) or placebo and were stratified by tumour origin, World Health Organization performance status and prior SSA treatment. There was a statistically significant 52% reduction in the relative risk of progression or death in favour of everolimus, with a clinically relevant 7.1-month prolongation of progression-free survival (PFS) compared with those who had taken placebo. In addition, everolimus was well tolerated and its established safety profile confirmed.

Over many years, peptide receptor radionuclide therapy (PRRT) using radiolabelled octreotide has been extensively used for the treatment of progressive NETs, and while individual results have been encouraging, there has been no formal assessment of such therapy. For most NETs, molecular-targeted radiation therapy involves the systemic administration of a radiolabelled peptide designed to target somatostatin receptors on tumour cells with high affinity and specificity.^15^ Over the past 15 years, PRRT with the radiolabelled somatostatin receptor agonists, such as ^90^Y-DOTATOC, ^177^Lu-DOTATATE and ^177^Lu-DOTATOC, have been successfully used to target metastatic and inoperable NETs.^15–16^ Now, Strosberg and colleagues have presented data from the NETTER-1 trial,^17^ a phase III multicentre, stratified, open, randomised, controlled trial evaluating the efficacy of ^177^Lu-DOTA^0^-Tyr^3^-Octreotate (^177^Lu-DOTATATE or Lutathera®[Advanced Accelerator Applications, Saint-Genis-Pouilly, France]) in patients with inoperable, somatostatin receptor-positive mid-gut NETs progressing on LAR (20–30 mg every 3–4 weeks). The trial recruited 230 patients from 35 sites in eight European countries and 15 centres in the US, with grade 1–2 mid-gut NETs. Patients were randomised to receive either four administrations of *Lutathera* (7.4 GBq every 8 weeks) plus their octreotide LAR or ‘high-dose’ octreotide LAR (60 mg every 4 weeks). PFS was evaluated via Response Evaluation Criteria In Solid Tumors (RECIST) criteria every 12 weeks. The median PFS was not reached for *Lutathera* (in more than 25 months of treatment) but was 8.4 months for the high-dose octreotide LAR group (p<0.0001), with the number of disease progressions/deaths 23 in the *Lutathera* group and 67 in the Octreotide LAR group. The estimated PFS in the *Lutathera* arm was 40 months. This phase III trial showed a statistically significant increase in PFS in patients with advanced mid-gut NETs treated with *Lutathera* providing a valid therapeutic option with minimal adverse events.

Finally, a significant problem in patients with mid-gut carcinoids is the carcinoid syndrome (CS) of principally diarrhoea and flushing. The symptoms of CS have been attributed, in part, to elevated levels of 5-hydroxytryptamine (5-HT) (serotonin) secreted by the tumour.^9,18^ In current practice, patients with CS are generally treated with long-acting SSAs which have an inhibitory effect on tumour secretion of serotonin and other neuropeptides into the systemic circulation. Many patients respond to SSAs initially, but some are refractory, while in others the response may be lost over time. In 2014 Kulke and coworkers published the results of a preliminary study to assess the safety of an orally active 5-HT synthesis inhibitor, telotristat etiprate (TE), compared with placebo in 23 patients with CS-related diarrhoea inadequately controlled by octreotide.^19^ TE acts by inhibiting tryptophan hydroxylase, the rate-limiting enzyme in the conversion of tryptophan to serotonin in NETs. Their preliminary results showed a proof-of-concept supporting the clinical benefit of TE across several endpoints, including decreased frequency of bowel movements (BM), decreased urinary 5-hydroxyindoleacetic acid (5-HIAA) levels and patient-reported relief of symptoms.^19^ A prospective, exploratory, dose-escalating 12-week, open-label, multicentre study of TE on 15 patients with metastatic, well-differentiated, NETs and CS showed improvement in CS with a 74% reduction in urinary 5-HIAA and a 43.5 % reduction in BM.^20^

At the ECC meeting, Kulke and colleagues presented the results of TELESTAR,^21^ a pivotal phase III global clinical trial evaluating the efficacy of TE in treating patients with CS inadequately controlled by SSA therapy. This trial included 135 patients with metastatic NETs and inadequately controlled CS (≥4 daily BMs on SSA therapy) who were randomly assigned to receive TE (250 or 500 mg) or placebo while continuing SSA over a 12-week double-blind period. The primary objective was met, with at least a 30 % reduction in BM frequency for ≥50 % of the time on study in 20 %, 44 % and 42 % on placebo, TE 250 mg three times daily (tid) and TE 500 mg tid, respectively (p≤0.040 for both TE doses versus placebo). Secondary objectives including changes in urinary 5-HIAA, cutaneous flushing episodes and abdominal pain were met for both dosage regimes, but reductions in flushing and abdominal pain were not statistically significant. Thus, TE appears to be a new approach to the treatment of the CS, which is generally safe and well tolerated, and represents a promising new class of therapy for patients who have metastatic NETs with CS not controlled by SSAs.

How do these new studies alter our approach to patients with NETs? It would seem that everolimus can retard the progression of all NETs regardless of their origin or functional status, although the tumours are generally stabilised rather than shrunk, and it is unclear as to when this therapy should be initiated and how robust is its long-term efficacy. For PRRT, we have long considered this may be of value: it is now reassuring to see this confirmed in a formal study, and it is hoped that *Lutathera* will soon become commercially available. However, the sequencing of such treatments, and the costs of their administration, remain problematic matters. Finally, it will be very useful to have TE added to our armamentarium of agents for CS-associated symptoms; while its therapeutic efficacy is perhaps not as major as one might have anticipated, the possible effects on avoiding or minimising mesenteric fibrosis and cardiac valvular dysfunction remains an exciting possible innovation.^22^

**CLARINET** – Study of Lanreotide Autogel in Non-functioning Entero-pancreatic Endocrine Tumours

**NETTER-1** – A Study Comparing Treatment With 177Lu-DOTA0-Tyr3-Octreotate to Octreotide LAR in Patients With Inoperable, Progressive, Somatostatin Receptor Positive Midgut Carcinoid Tumours

**PROMID** – Placebo Controlled, Double Blind, Prospective, Randomized Study on the Effect of Octreotide LAR in the Control of Tumor Growth in Patients with Metastatic Neuroendocrine Midgut Tumors

**RADIANT-2** – Everolimus and Octreotide in Patients With Advanced Carcinoid Tumor

**RADIANT-3** – Efficacy and Safety of Everolimus (RAD001) Compared to Placebo in Patients With Advanced Pancreatic Neuroendocrine Tumors

**RADIANT-4** – Everolimus Plus Best Supportive Care vs Placebo Plus Best Supportive Care in the Treatment of Patients With Advanced Neuroendocrine Tumors (GI or Lung Origin)

**TELESTAR** – Telotristat Etiprate for Somatostatin Analogue Not Adequately Controlled Carcinoid Syndrome
